# Supplementary material for: Effect of predation risk and ectoparasitic louse flies on physiological stress condition of the red-tailed tropicbird (Phaethon rubricauda) from Rapa Nui and Salas & Gómez islands
Source: PeerJ. 2020 Jul 8;8:e9088. doi: 10.7717/peerj.9088 (PMC7353918; doi:10.7717/peerj.9088)
Supplement: Supplemental Information 1 [file peerj-08-9088-s001.docx]

**Table S1.** Bill, skull, wing and tarsus length and weight of red-tailed tropicbird adults assessed between August 2016 and June 2017 in Rapa Nui and Salas & Gómez islands.

|  | Mean | SD | Mean | SD | Mean | SD |
| --- | --- | --- | --- | --- | --- | --- |
| Rapa Nui | Total n = 26 | | Female n = 15 | | Male n = 11 | |
| Bill (mm) | 66,8 | 2,6 | 66,8 | 2,8 | 66,7 | 2,5 |
| Skull (mm) | 133,4 | 3,9 | 133,7 | 3,7 | 132,9 | 4,5 |
| Wing (mm) | 340,9 | 9,5 | 338,7 | 7,7 | 343,9 | 11,2 |
| Tarsus (mm) | 36,1 | 2,1 | 35,6 | 2,4 | 36,7 | 1,5 |
| Weight (g) | 826,2 | 56,2 | 824,3 | 66,2 | 828,6 | 41,7 |
| Salas & Gómez | Total n = 25 | | Female n = 12 | | Male n = 13 | |
| Bill (mm) | 64,20 | 3,3 | 62,8 | 3,5 | 65,5 | 2,4 |
| Skull (mm) | 130,6 | 3,6 | 128,9 | 3,3 | 132,1 | 3,1 |
| Wing (mm) | 339,8 | 8,9 | 340,0 | 8,9 | 339,7 | 9,4 |
| Tarsus (mm) | 35,9 | 2,5 | 34,9 | 2,1 | 36,8 | 2,5 |
| Weight (g) | 824,2 | 82,9 | 805 | 79,9 | 841,9 | 84,7 |
